# Human–Wildlife Conflict in Bardia—Banke Complex: Patterns of Human Fatalities and Injuries Caused by Large Mammals

**DOI:** 10.1002/ece3.70395

**Published:** 2024-10-08

**Authors:** Umesh Paudel, Rabin Bahadur KC, Rabin Kadariya, Ajay Karki, Bishnu Prasad Shrestha, Shyam Kumar Shah, Naresh Subedi, Shyam Kumar Thapa

**Affiliations:** ^1^ National Trust for Nature Conservation Lalitpur Nepal; ^2^ Department of National Parks and Wildlife Conservation Kathmandu Nepal; ^3^ Department of Zoology and Physiology University of Wyoming Laramie Wyoming USA; ^4^ Wildlife Ecology and Conservation Wageningen University and Research (WUR) Wageningen The Netherlands; ^5^ Agriculture and Forestry University Chitwan Nepal; ^6^ Zoological Society of London Nepal Office Kathmandu Nepal

**Keywords:** Bardia—Banke Complex, human–wildlife conflict, large mammals, spatial and temporal patterns

## Abstract

Human fatalities and injury from wildlife attacks often result in a negative attitude toward conservation. This research was undertaken to investigate the patterns and conflict‐causing factors of human killing and injury by large mammals, especially by Asian elephant (*Elephas maximus*), common leopard (*Panthera pardus*), and Bengal tiger (*Panthera tigris tigris*) in the Bardia—Banke Complex of western Nepal. We collected human death and injury records caused by wildlife in the Bardia—Banke Complex between 2019 and 2023, based on relief applications submitted by the victim's family. Additionally, camera trap monitoring was conducted following incidents of human–tiger and human–leopard conflicts. A total of 76 incidents involving human casualties and injuries were considered for analysis. Incidents of livestock depredation, crop raiding, and property damage were excluded from the analysis. Most of the attacks on humans were caused by tigers (75%), followed by elephants (16%) and leopards (9%). Almost all incidents occurred in daytime (97%). The highest number of conflicts were recorded in 2021, with 20 incidents. Most of the cases (84%) occurred within 1 km of forest edge. Khata corridor and the western side of the Bardia National Park, i.e., Karnali River corridor, were identified as high‐conflict areas. The primary causes of the conflict manifested in cattle grazing (28%), grass cutting (28%), firewood collection (11%), fishing (8%), vegetable collection (5%), sand collection (4%), during rescuing friends (3%), grazing captive elephants (3%), highway rides (3%), sleeping in Chaupadi Goth (3%), walking nearby forest areas (3%), playing nearby forest areas (1%), while feeding pig (1%), and working in agricultural lands (1%). To promote human–wildlife coexistence, community‐based patrols (33%), habitat restoration (26%), electric fencing (26%), and insurance (7%) were identified as the preferred strategies. Therefore, we recommend that stakeholders and concerned bodies increase awareness among local community about the use of forest resources, wildlife behavior, and human–wildlife conflict mitigation strategies.

## Graphical abstract

This research investigates the patterns and conflict‐causing factors related to human fatalities and injuries caused by large mammals in the Bardia—Banke Complex of western Nepal.

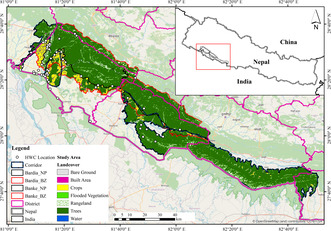

## 1 Introduction

### 1.1 Background

Conflict between humans and wildlife is among the most widespread and pressing challenges to wildlife conservation (Nyhus, 2016). The continuous expansion of human population, driven by the prioritization of development and the spread of settlements, has resulted in a global reduction in suitable wildlife habitats. As a result, wildlife is confined within protected areas worldwide (Strassburg et al. 2020). Yet, in many parts of the world, wildlife, especially that require extensive home range, venture out from the protected areas where they frequently come into conflict with people residing adjacent to the protected areas (Prins, Liefting, and De Jong 2022). Human–wildlife conflicts (HWC) refer to the negative interaction between wild animals and humans, with negative consequences on both sides (IUCN SSC Human–Wildlife Conflicts Task Force 2020). These conflicts occur when wildlife compromises the achievement of human goals or when human interests and actions come at the cost of the survival of wildlife (Madden 2004). Such conflicts occur in a variety of forms including livestock depredation, crop loss, property damage, human injuries and casualties, and retaliatory killings of wildlife (Gurung et al. 2008). Conflicts become extremely controversial and problematic when endangered and protected wild species are involved in human–wildlife conflict (Acharya et al. 2016). As local communities refuse to accept wildlife‐caused conflicts due to the life‐threatening nature of wildlife attacks, they often retaliate by killing the animals involved (Treves and Bruskotter 2014).

In Nepal, people are attacked by large mammal species such as common leopard (*Panthera pardus*), elephants (*Elephas maximus*), Himalayan black bears (*Ursus thibetanus*), rhinoceros (*Rhinoceros unicornis*), sloth bears (*Melursus ursinus*), snow leopards (*Panthera uncia*), and tigers (*Panthera tigris*), but there is little discussion about the patterns of fatalities and injuries caused by wildlife or underlying temporal dynamics of HWC (Acharya et al. 2016; DNPWC 2022; Woodroffe 2000). Elephants, tigers, and leopards are the major conflict‐causing wild animals in Nepal and India, reported to cause extensive damage to human lives, livestock, crops, and property (Acharya et al. 2016; Naha, Sathyakumar, and Rawat 2018).

Protected areas that contain endangered species such as tigers, rhinoceros, and elephants in lowland Nepal are within human‐dominated landscape (Dinerstein et al. 2007). The local communities living adjacent to these important protected areas collect fodder, firewood, and various forest products that are important sources for their subsistence livelihoods (Leclerq et al. 2019). Until 2019, very few cases of human casualties caused by wildlife were recorded in Bardia National Park (BNP) and Banke National Park (BaNP). However, since 2019, HWC, particularly human–tiger conflict, has been trending high (Kadariya et al. 2023; Rauniyar 2021). With the highest tiger density in Nepal, BNP has witnessed an increase in human–tiger conflicts in recent years. However, the underlying causes remain unclear. Human–tiger conflicts are likely to occur where human and tiger habitats overlap, particularly along the edges of the national park (Woodroffe and Ginsberg 1998) or in a prime habitat when people venture into the core area to collect forest resources. Likewise, human–leopard conflict has increased rapidly in fringe areas of protected areas that hold tigers (Dhakal et al. 2023; Sijapati et al. 2021; Subedi et al. 2020). With the increasing tiger density within the core areas of protected areas, leopards are likely displaced and restricted to the fringe area near the human settlement (Odden, Wegge, and Fredriksen 2010; Rayan and Linkie 2016), leading to the human–leopard conflict. Similarly, elephants are also considered the major conflict‐causing animals in the lowland landscape of Nepal (Ram et al. 2021). Attack on humans by elephants, loss of crops, and damage to property are the major forms of human–elephant conflict, affecting socioeconomically marginalized communities living close to the fringe areas (Ram et al. 2021).

HWC leads to direct and indirect impacts on local communities and the species associated with the conflict (Sampson et al. 2021). In 2021 alone, a total of 12,672 HWC cases have been recorded in the protected areas of Nepal, out of which 58 people lost their lives, 116 were severely injured (severe injury: includes injuries that are significant and may have long‐lasting effects, e.g., permanent disability, loss of limbs/hands/eyes, or other serious health consequences), and 72 were slightly injured (slight injury: includes injuries that are less severe and typically do not have long‐term consequences; examples might include minor cuts, bruises, or sprain) (DNPWC 2022). In the fiscal year 2021, the Government of Nepal distributed a total amount of USD 1.2 million as compensation (DNPWC 2022). Specifically, in BNP and BaNP, a total of USD 160,000 and USD 86,000 were distributed in the community as compensation for HWC (DNPWC 2022). In addition, numerous efforts are in place to reduce HWC, for instance, the installation of electric/solar fences, the creation of physical barriers, the promotion of alternative crops, the promotion of predator‐proof corrals, etc. (DNPWC 2022; Hudu et al. 2017; Lamichhane et al. 2019). Despite these efforts, HWC is increasing in Nepal and has direct negative implications for the well‐being of HWC‐affected households (Meyer and Börner 2022), which might reduce social tolerance and may provoke retaliatory killings impacting the conservation of endangered species like tigers and elephants (Karanth, Gupta, and Vanamamalai 2018; Leslie et al. 2019).

Previous studies about human–wildlife conflict interaction in Bardia and Banke National Parks and their buffer zone focused either on single species (Bhattarai 2009; Prins, Liefting, and De Jong 2022; Upadhyaya et al. 2018) or solely on people's perception (Shahi et al. 2023), but comprehensive analyses of human–wildlife conflicts over a longer time span along with post‐analysis of each case using camera traps remain unreported. Thus, in our study, we present a comprehensive analysis of human–wildlife conflict (focusing on human casualties and injuries) around Bardia and Banke National Parks and their associated forest area during a time span of 5 years (2019 to 2023) to assess the (1) spatial and temporal trends related to human fatalities and injuries resulting from interactions with these large mammals and (2) reason behind each conflict case.

## 2 Methods and Methodology

### 2.1 Study Area

We collected HWC data from two national parks (Bardia National Park—BNP and Banke National Park—BaNP) and three corridors (viz., Karnali River corridor, Khata corridor, and Kamdi corridor), hereinafter termed as Bardia—Banke Complex (BBC). BNP was established in 1976 as Royal Karnali Wildlife Reserve, and later in 1988, it was renamed Royal Bardia National Park, which was later renamed Bardia National Park in 2010, and BaNP was established in 2010. Geographically, BNP is located at 28^o^ 15′ to 28^o^ 35.5′ N and 80^o^ 10′ to 81^o^ 45′ E (BNP 2022), and BaNP is located between 27^o^ 58′13″ to 28^o^ 21′26″ N and 81^o^ 39′29″ to 82^o^ 12′19″ E (BaNP 2022).

The study area (BBC) is administratively stretched along three provinces, i.e., Lumbini, Karnali, and Sudurpaschim provinces of Nepal. BaNP and BNP are adjacently connected, with BNP connecting to Katarniaghat Wildlife Sanctuary in India in the south via the Khata corridor and BaNP connecting to Suhelwa Wildlife Sanctuary in India via the Kamdi corridor (BaNP 2022; Bhatt et al. 2023; BNP 2022).

The BBC is characterized by a subtropical to temperate climate and dominant vegetation types, including Sal (*Shorea robusta*) and mixed hardwood forest at low altitudes and pine forest at higher altitudes (MFSC 2015). The study area holds more than 60 species of mammals, including charismatic species like tigers, rhinoceros, elephants, 513 species of birds, and 42 species of herpetofauna (BaNP 2022; BNP 2022).

The BBC lying within Terai Arc landscape (TAL) is an important habitat for tigers (Fitzmaurice et al. 2021; Subedi et al. 2020). BNP is the largest national park in the plains (Terai), characterized by high human population density (BNP 2022; National Statistics Office 2021). Similarly, the newly established BaNP also features a good population of large mammals (BaNP 2022). BBC holds the second largest tiger population in Nepal (150), with the highest tiger density, i.e., 7.15 (SD 0.38) in BNP and 0.97 (SD 0.12) in BaNP (DNPWC and DFSC 2022). Also, the complex supports the highest number of Asian elephants in Nepal, i.e., 120–140 (Ram et al. 2021). The common leopard exists as a competitive predator along with other carnivores in the BBC (Sharma, Chettri, and Wangchuk 2021; Stein et al. 2019), with leopards sharing habitat and prey with tigers (Sijapati et al. 2021; Tamang and Baral 2008).

The study area comprises people of different ethnicities from the indigenous Tharu community, Brahmin, Chhetri, Magar, Tamang, Majhi, and Gurung who are living within the buffer zone having very low economic conditions (CBS Nepal 2022). Human population density is high in much of Terai (about 400 persons/km^2^), and land is a scarce commodity (Population Education and Health Research Centre 2016). The local economy is almost completely based on farming with extensive irrigation systems (Prins, Liefting, and De Jong 2022). In the study area, farmers are at severe risk (45%) of undernourishment, based on a minimum requirement of dietary energy consumption of 1,810 kcal/person/day (Joshi et al. 2010).

### 2.2 Methods

To extract the relevant conflict data, we first obtained permission from the Banke and Bardia National Parks and also National Trust for Nature Conservation (NTNC) authorities to access their database. We collected data on wildlife attacks (common leopard, elephant, and tiger) on humans reported to BNP and BaNP authorities during 2019–2023. Additionally, we also assessed the camera trapping monitoring data, which has been deployed by NTNC after a conflict case to identify the conflict‐causing reason. For each conflict event, we attempted to document the following data: (1) type of conflict (death or injury); (2) species involved; (3) time of incident (year, month, and season) (Winter: December–February; Spring: March–May; Summer: June–August; Autumn: September–November); (4) location of conflict (forest, farmland, or home); (5) whether the conflict was inside or outside the existing forest area; and (6) what the victim was doing when attacked?

A total of 24 key informant interviews (KIIs) were conducted that included park officials (game scouts = 8, rangers = 2, and officers = 2), representatives from Bardia Conservation Program of National Trust for Nature Conservation (*n* = 4), member of Anti‐Poaching Units (*n* = 5), and Buffer Zone User Committee members (*n* = 3). The purpose of these KIIs was to identify mitigation measures to address the increasing human–wildlife conflict. These interviews helped us to gain a deeper understanding of the root cause of conflicts and the effectiveness of current conflict management strategies in BNP and BaNP.

We classified each incident as either a casualty (coded as 1) or injury (coded as 0). Descriptive summaries of conflict incidents, including yearly, monthly, and seasonal wildlife attacks on humans were calculated using the Pivot table function in MS Excel 365, and statistical analyses were done in R (R Core Team 2022). Chi‐square tests of independence or, in cases where there were a small number of observations, Fisher's exact tests were applied to compare the frequency of attacks (fatalities and injuries) by each wildlife species (elephant, leopard, and tiger) in relation to time (year, season, and month), location (forest, farmland, and home) and whether they were inside the forest area (park boundary) (Acharya et al. 2016). Kernel density maps were used to estimate the intensity of the conflict within the BBC using Arc GIS 10.5 (Fleming et al. 2015).

## 3 Results

### 3.1 Human Deaths and Injury

**FIGURE 1:**
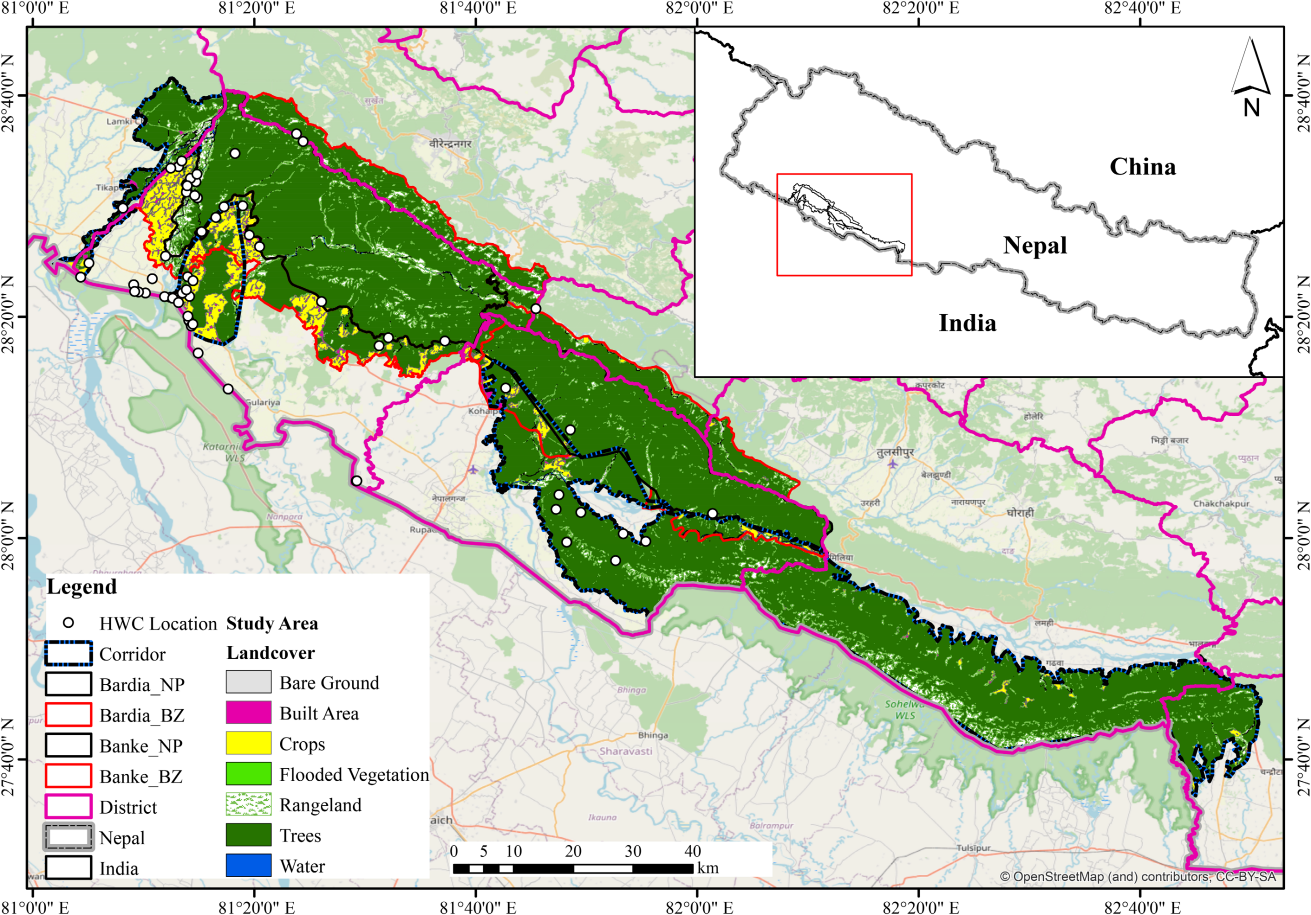
Landcover over the Banke and Bardia National Parks along with three corridors, i.e., Karnali River, Khata, and Kamdi corridors.

BBC has recorded a total of 76 wildlife attacks on humans between 2019 and 2023, with an annual average of 15.2 (SD 4.08). Similarly, we found an average of 4.8 human injuries (SD 2.8) and 10.8 human casualties (SD 4.9) (Figure 1). Out of these cases, a maximum of 75% (*n* = 57) cases were caused by tiger, followed by 16% (*n* = 12) by elephants and 9% (*n* = 7) by leopard (Table 1). Of the 76 victims, 58% (*n* = 44) were male and 42% (*n* = 32) were female (*χ* ^2^ = 3.169, df = 1, *p* > 0.05). Age groups between 31 and 55 were the most affected, with 45% (*n* = 34) of human attacks falling within this category. Likewise, 29% (*n* = 22) of cases were associated with the age group above 55 years old, followed by the age group between 16 and 30, < 14 years old by 22% (*n* = 17) and 4% (*n* = 3), respectively.

**TABLE 1:** Total number of human attacks caused by elephant, leopard, and tiger in Bardia—Banke Complex and its associated forests for the period of 2019–2023.

Species	Injured	Killed
Elephant	0	12
Leopard	4	3
Tiger	18	39
Total	**22**	**54**

### 3.2 Temporal Pattern of Human Injuries and Fatalities

**FIGURE 2:**
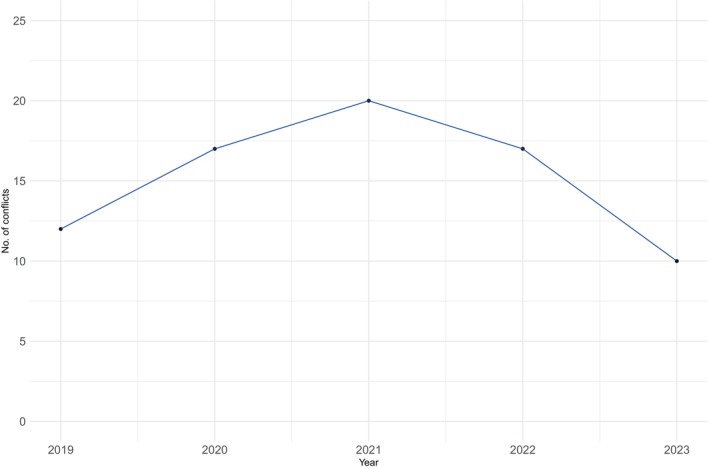
Frequency of wildlife attack to human from 2019 to 2023.

The wildlife attack on humans seems to be in a decreasing trend. The number of conflicts in 2019 was 16% (*n* = 12), which increased to 22% (*n* = 17) in 2020. Further, in 2021, the number of conflicts spiked to 26% (*n* = 20) as the highest, and in 2022, it decreased to 22% (*n* = 17), and in 2023, it reached to 13% (*n* = 10) (*χ* ^2^ = 9.7746, df = 4, *p* < 0.05, Figure 2).

**FIGURE 3:**
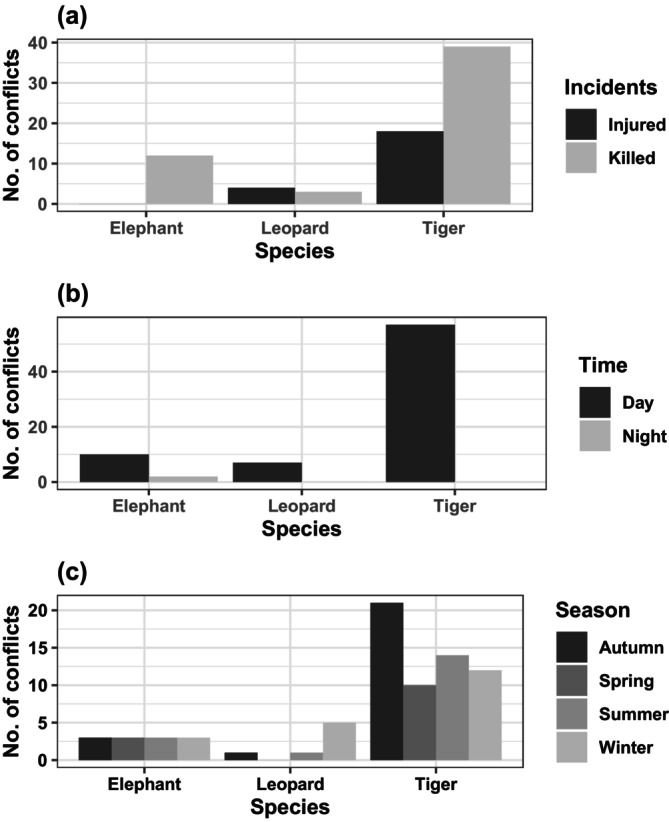
Frequency of wildlife conflict in the BBC. (a) Number of incidents with respect to human injury or kill (b) with respect to time (day or night) and (c) with respect to season.

**FIGURE 4:**
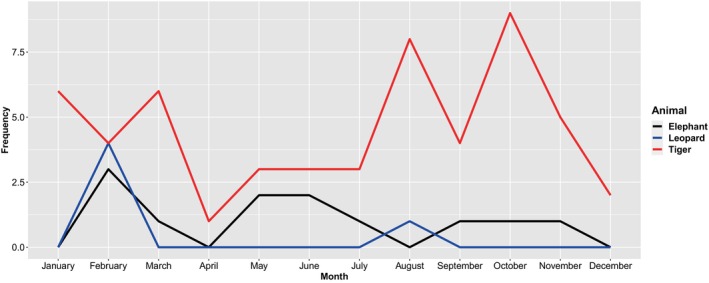
Monthwise trend of attack by elephant, leopard, and tiger from 2019 to 2023.

Almost all recorded cases have occurred in the daytime 97% (*n* = 74, out of 76 cases), and only a few cases 3% (*n* = 2, out of 76 cases) in the nighttime (*χ* ^2^ = 68.221, df = 1, *p* < 0.05), especially associated with conflicts with tigers and leopards. Only, 3% (*n* = 2) happened during the nighttime which is caused by the elephant (Figure 3). The highest number of attacks, 33% (*n* = 20), was recorded in the autumn season, followed by winter and summer with 26% (*n* = 20) and 24% (*n* = 18), respectively (Figure 4). Conflict in spring was comparatively lower, at 17% (*n* = 13) (*χ* ^2^ = 3.894, df = 3, *p* > 0.05).

We observed that the human–elephant conflict was high in February, May, and June (Figure 4). Similarly, the frequency of human–leopard conflict also peaked in February, followed by a sharp decline, with a minor resurgence in August. In contrast, human—tiger conflict displayed a different pattern, with relatively high and consistent conflict levels early in the year, a peak in October, and a subsequent decline toward December (Figure 4).

### 3.3 Spatial Pattern of the Occurrence of Human Injuries and Fatalities

**FIGURE 5:**
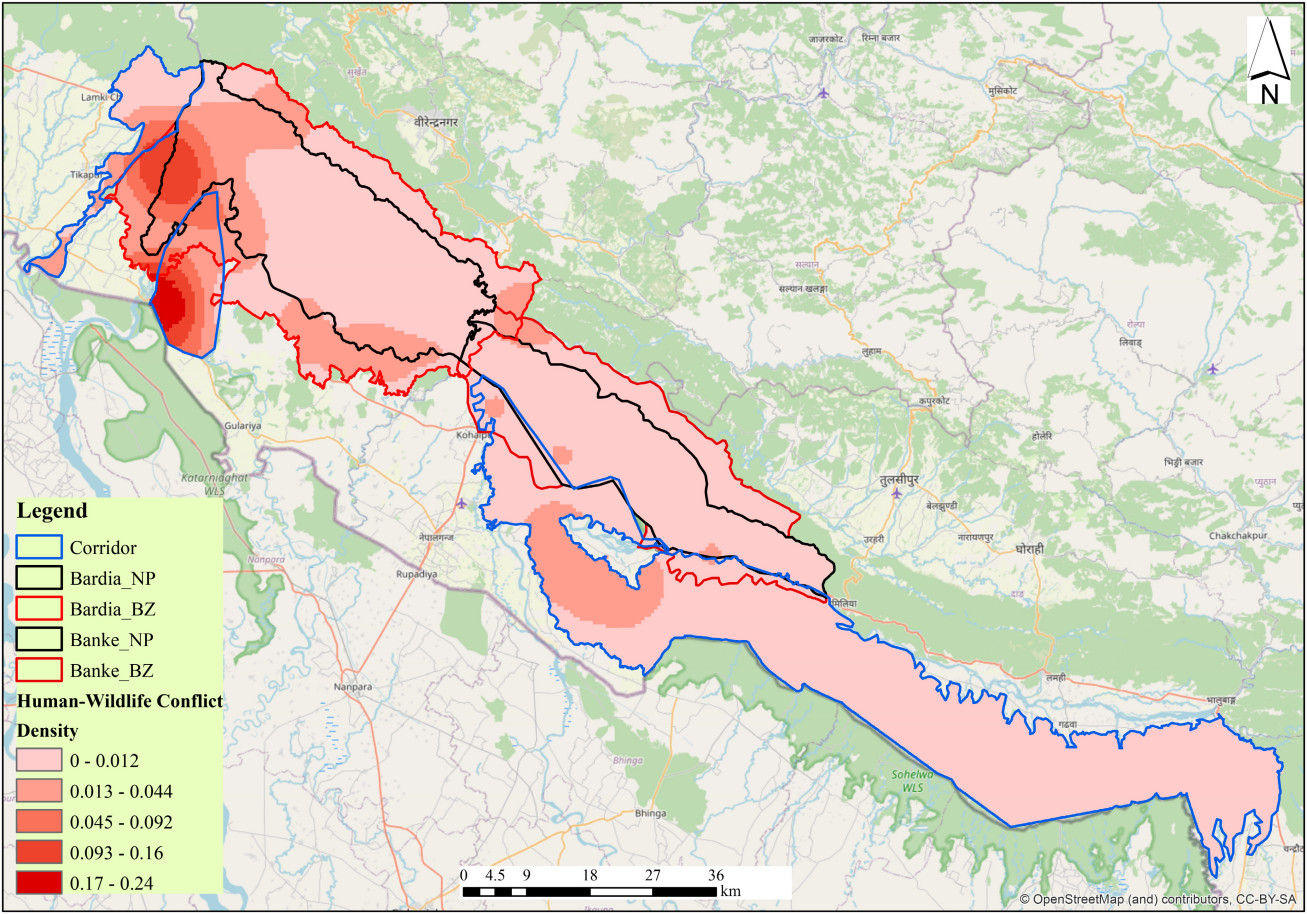
Spatial distribution of human–wildlife conflict in the Bardia—Banke Complex.

**FIGURE 6:**
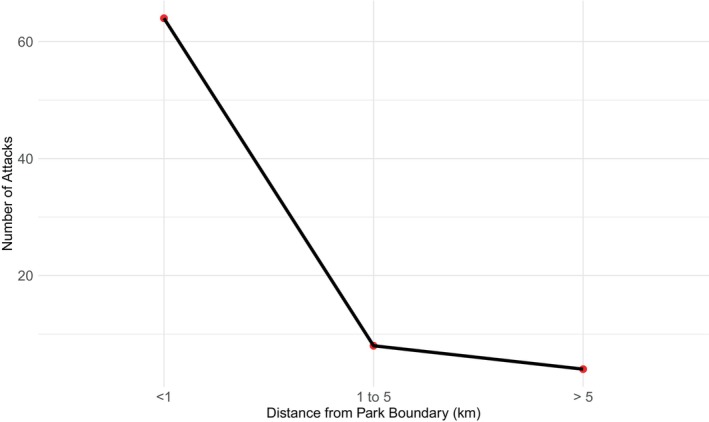
Distance between the conflict location and forest area.

Out of the total cases of human–tiger conflict recorded in the BBC, 13% (*n* = 10) of cases were recorded from BaNP, of which two cases were of human injuries and eight cases were of human death. All the tiger conflict cases from BaNP were recorded only from the buffer zone forest area, especially from Khairi area. Similarly, in the BNP, the Khata corridor and Karnali River corridor were categorized as high‐conflict areas (Figure 5). The distance to the forest area and park boundary is highly significant to the conflict cases (*χ* ^2^ = 88.842, df = 2, *p* < 0.05), as 84% of cases were recorded nearer the national park, with the distance less than 1 km (Figure 6).

## 4 Causes for the Conflict

**FIGURE 7:**
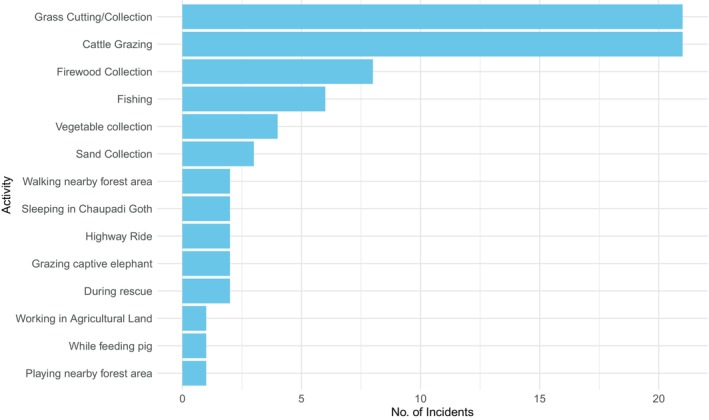
Reasons associated with the human–wildlife conflict incidents.

Grass collection and cattle grazing were the major underlying factors leading to the conflict with wild animals (Figure 7), with 21 cases reported (*χ* ^2^ = 113.74, df = 13, *p* < 0.05). Other activities that resulted in conflict included firewood collection (8 cases), fishing (6 cases), vegetable collection (4 cases), sand collection (3 cases), during rescuing other friends in the jungle (2 cases), while sleeping outside the house in a cowshed (*Goth*) as a *Chaupadi* (2 cases) (Chaupadi Pratha is an extreme form of menstrual taboo where women are considered untouchable and are isolated during their menstruation and childbirth) (Joshi 2022), walking nearby the forest area (2 cases), grazing captive elephant (2 cases), riding through the national park via highway (2 cases), playing nearby forest area (1 cases), and working in agricultural land (1 cases); one case occurred when an adult male was feeding a pig (Figure 7).

**FIGURE 8:**
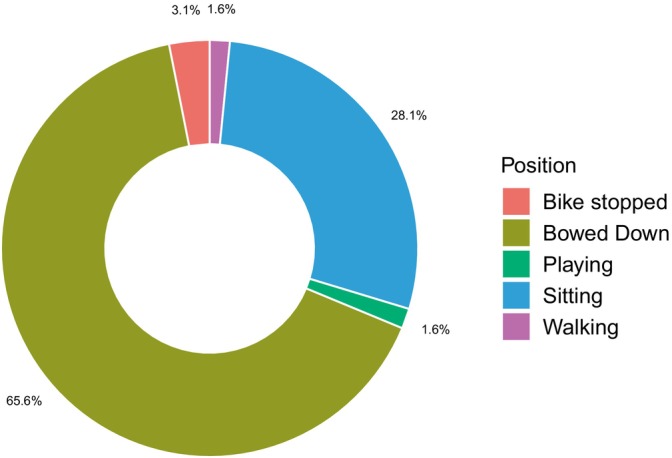
Position of victims when the carnivore attacked.

Also, out of the total cases, 64 were caused by carnivores, i.e., tigers and leopards. Therefore, from the KII and postmonitoring of the cases, we realized that during the attack, maximum victims bowed down (94% of the cases) like a four‐footed animal and/or sitting on the ground, looking smaller and similar to a prey (*χ* ^2^ = 99.594, df = 4, *p* < 0.05) (Figure 8).

### 4.1 Confilict Mitigation and Promoting Coexistence

A total of USD 459,639.7 has been distributed as a relief fund from the Government of Nepal for the 76 conflict cases. The KII responses provide insights into preferred strategies for mitigating human–wildlife conflicts. Community‐based patrols were the most favored, with 9 out of 24 respondents (33%) recognizing their effectiveness in local monitoring and rapid response to wildlife encounters. Similarly, habitat restoration and use of electric fencing received 26% (*n* = 7) and 22% (*n* = 6) response to contribute to the mitigation of HWC. Interestingly, only 7% (*n* = 2) respondents regarded crop/livestock insurance as the mitigation factor, though it provides essential financial protection for farmers.

Similarly, on effective strategies for communities to coexist with wildlife, education and awareness program emerged as the top strategy, supported by 67% (*n* = 16) of respondents. Additionally, 21% (*n* = 5) endorsed ecotourism and 13% (*n* = 3) emphasized the need to enhance relief distribution schemes for better coexistence.

## 5 Discussion

Our findings highlight the spatial and temporal trends of human fatalities and injuries resulting from encounters with large mammals, providing valuable insights into future conservation requirements. In our study, we specifically focused on tigers, elephants, and leopards, emphasizing the patterns and determinants of HWC in the BBC. Our results reveal that human–tiger conflict poses the most pressing challenge, particularly for the conservation of endangered tigers.

The intensity of human–wildlife conflict was found to increase in proximity to the national park boundaries. This aligns with the findings by Gurung et al. (2008), who recorded a similar pattern from Chitwan National Park where the maximum cases of conflict were within 1 km of the forest edge. Similarly, Bhatta and Joshi (2020) recorded a similar result from Shuklaphanta National Park. Communities residing near forest areas depend on agriculture for their livelihoods, which in turn relies on adjacent natural resources. This reliance exacerbates human—wildlife interactions and conflicts (Nepal 2002). Individuals within the active age groups (31–55 years), who are primarily responsible for household activities like fodder collection, grass cutting, and livestock grazing, may be at a greater risk of encountering wildlife due to their direct engagement and frequent visits to forest areas.

Our study confirms that wildlife attacks peak during daylight hours. Although, Fitzmaurice et al. (2021) mentioned that tigers and leopards are typically inactive around midday, they can still exhibit defensive behavior if surprised, corroborating our findings. Mostly, tiger and leopard attacks appeared to be opportunistic. Similar patterns of attacks have been recorded in Bangladesh and India (Kabir 2019; Mathur 2014). A higher number of conflict cases were recorded during the winter season, which may be attributed to the fact that community people are comparatively free with not much work in the agricultural field (Baral et al. 2021) compared to the summer season. Consequently, more people are likely to visit national parks and forest areas to collect firewood, fodder, and wild vegetables, increasing the likelihood of wildlife encounters, resulting in increased cases of conflicts in the winter season.

Postmonitoring of conflict cases revealed that conflicts occurred in forest areas while associated with forest resources like grass cutting, cattle grazing, firewood collection, fishing, sand collection, and vegetable collection, which corroborates with previous studies (Bombieri et al. 2018; Silwal et al. 2017). These findings highlight that human behavior, particularly resource collection practices, plays a significant role in triggering conflicts. Previous studies also highlighted that human behavior toward wildlife determines the severity of human–wildlife conflict (Acharya et al. 2016; Kadariya et al. 2023). Fitzmaurice et al. (2021) also found that livestock herding and fodder collection were common activities leading to human–tiger conflict in the BBC, aligning with Silwal et al. (2017), who reported that 45% of conflict incidents in Chitwan National Park during 2003–2013 occurred during natural resource collection. Only two cases recorded in the nighttime were caused by elephants, and one case was caused by tigers. Our study showed no specific pattern for months and seasons. However, we documented the maximum cases of conflict associated with elephants in February, possibly due to elephants being attracted to wheat crops during this time. Pant et al. (2016) and Silwal et al. (2017) also observed that most attacks by elephants occurred at night in the winter season around February, while Ram et al. (2021) observed elephant attacks peaked during September—December.

This study is the first to highlight, through post‐human–tiger conflict case analyses, the significance of victim posture during human–tiger conflict incidents. Our findings show that, apart from a few cases involving motorbikes and morning walks, all other victims were bowing down to the ground while collecting forest resources. This novel observation suggests that the act of bowing down, particularly in the context of resource gathering, may unintentionally signal vulnerability to tigers, as a lowered posture can resemble prey behavior, potentially triggering a predatory response from tigers, contributing to the occurrence of conflicts. This insight is crucial for understanding human–tiger conflict mitigation strategies. By incorporating these findings into conservation and safety measures, more effective approaches can be developed to minimize conflict, ensuring the safety of local communities, and promoting coexistence with these magnificent yet potentially dangerous predators in their natural habitats.

Our study showed that human–tiger conflict is severe in the BBC, unlike other studies where elephant is considered to be the major conflict‐causing animal (Acharya et al. 2016). This finding is crucial for park management to conserve and maintain the increasing tiger population in the national parks that hold tigers. Nepal was one of the first countries to nearly triple the tiger population by 2022 after its global commitment of doubling the tiger population in the wild in 2010 in Russia. However, increasing human–tiger conflict cases (54 cases within 5 years, with 39 cases of human fatality only in the BBC) presents a serious challenge for the long‐term conservation of tigers in Nepal. Despite the rising cases of human–tiger conflict incidents, many local community members remain unaware of the risk posed by the growing number of tigers in the national parks. Informal discussion with the local people revealed that they do not perceive the risk as significantly higher than that it was a decade ago, when there were fewer tigers in the area. This lack of awareness highlights the need for stronger outreach and education efforts to mitigate conflict and protect both human and wildlife population.

## 6 Conservation Implications

Human–wildlife conflict is on an increasing trend, and most of the conflict cases in the BBC occurred in forests, mainly when people enter forests to collect forest resources. Decreasing the forest dependency of economically backward people by providing alternative livelihood opportunities would help decrease the encounter of wildlife with people. More importantly, the behavior and attitude of people are the crucial aspects to consider while devising HWC mitigation measures. Current efforts to minimize HWC include construction of physical barriers such as electric fences, concrete fences, game‐proof fences, and biofences (BaNP 2022; Bhattarai 2009; Bhattarai, Wright, and Khatiwada 2016; Bhattarai et al. 2019; BNP 2022). All these measures are targeted specially to stop larger mammals such as elephants and rhinoceros from entering into settlements (Acharya et al. 2016; Hudu et al. 2017). However, from this study, it is observed that human–tiger conflict is the most severe in the BBC, posing serious challenge to wildlife conservation. Our study reported that most victims were attacked while trespassing into the forest area to collect forest resources, indicating that their outdoor activities make them more vulnerable to attack, which is consistent with the results of previous studies (Dhanwatey et al. 2013; Gurung et al. 2008; Nyhus and Tilson 2004; Pant et al. 2016; Silwal et al. 2017).

Mitigating HWC requires a multifaceted approach. Reducing forest dependency through alternative livelihood strategies (e.g., reducing grazing, increasing off‐farm household income, etc.), enhancing conflict mitigation infrastructure (e.g., electric fence, mesh‐wire fence, and biofence), and promoting behavior change through public awareness campaigns could collectively contribute to minimize HWC. We recommend indulging with the socioecological aspect of conservation to increase the adaptation and resilience capacity of the local community in the conservation of endangered species, ensuring the capacity of the local community to adapt and respond effectively to conservation challenges. This will ensure coexistence through effective wildlife management (ecological aspect) and mechanisms to reduce human–wildlife conflicts (social aspect).

## Author Contributions

**Umesh Paudel:** conceptualization (lead), data curation (equal), formal analysis (equal), investigation (equal), methodology (equal), validation (equal), visualization (equal), writing – original draft (lead). **Rabin Bahadur KC:** conceptualization (equal), data curation (equal), formal analysis (lead), investigation (equal), methodology (equal), writing – original draft (equal), writing – review and editing (lead). **Rabin Kadariya:** conceptualization (equal), data curation (equal), investigation (equal), supervision (lead), writing – review and editing (equal). **Ajay Karki:** investigation (equal), methodology (equal), supervision (equal), writing – review and editing (equal). **Bishnu Prasad Shrestha:** investigation (equal), methodology (equal), supervision (equal), validation (equal). **Shyam Kumar Shah:** investigation (equal), methodology (equal), supervision (equal), validation (equal). **Naresh Subedi:** investigation (lead), methodology (equal), supervision (equal), validation (lead), writing – review and editing (equal). **Shyam Kumar Thapa:** formal analysis (equal), methodology (equal), supervision (equal), validation (equal), writing – original draft (equal), writing – review and editing (equal).

## Conflicts of Interest

The authors declare no conflicts of interest.

## Data Availability

Data associated with this manuscript can be accessed at the Dyrad data repository (https://datadryad.org/stash/share/kzxnhfBvLUfDQxtgAl5LFDNzYy_NkIcSkdL7jeFjrRo).
